# Openness and Entrepreneurial Performance During COVID-19 Pandemic: Strategic Decision Comprehensiveness as an Inconsistent Mediator

**DOI:** 10.3389/fpsyg.2021.806756

**Published:** 2022-01-13

**Authors:** Weiqi Mu, Jie Xu, Fugui Li, Siying Li, Xue Li, Mingjie Zhou

**Affiliations:** ^1^CAS Key Laboratory of Mental Health, Institute of Psychology, Chinese Academy of Sciences, Beijing, China; ^2^Department of Psychology, University of Chinese Academy of Sciences, Beijing, China; ^3^College of Education, Anyang Normal University, Anyang, China

**Keywords:** COVID-19, openness, entrepreneurial performance, strategic decision comprehensiveness, inconsistent mediation

## Abstract

The COVID-19 pandemic severely hit small and micro-businesses. In the face of the impact of the pandemic, how to help entrepreneurs, especially small- and micro-businesses that are more sensitive to the impact of the pandemic, make decisions to reduce losses has become an issue worth paying attention to. From the perspective of personality approach, this article studied openness, which is the strongest predictor of entrepreneurial performance among the big five personality traits, and explored the impact of entrepreneurs’ openness on entrepreneurial performance during the COVID-19 pandemic, as well as the inconsistent mediating role of strategic decision comprehensiveness on entrepreneurial performance. An online questionnaire survey was conducted among 238 entrepreneurs of small- and micro-businesses when China was recovering from the pandemic and starting to resume work and production (February 18 – February 26, 2020). Entrepreneurial performance during the COVID-19 pandemic was measured by comparing the business conditions before and after the pandemic. The results showed that entrepreneurs’ openness positively impacted strategic decision comprehensiveness and entrepreneurial performance during the COVID-19 pandemic. Among the two competing hypotheses proposed by summarizing previous research, the results supported that strategic decision comprehensiveness negatively affected entrepreneurial performance. It indicated that entrepreneurs who tend to collect and analyze information extensively and then make decisions during the pandemic could not seize opportunities and improve their entrepreneurial performance. The results further supported that strategic decision comprehensiveness was an inconsistent mediator between openness and entrepreneurial performance, showing that entrepreneurs with low openness can also reduce the loss of entrepreneurial performance during the pandemic by making incomplete but rapid strategic decisions. This study found that the openness of entrepreneurs had a positive impact on strategic decision comprehensiveness for the first time and provided more empirical evidence for the negative effect of strategic decision comprehensiveness on entrepreneurial performance in the context of information uncertainty and unanalyzable situations. The inconsistent mediating effect of strategic decision comprehensiveness revealed in this study also has practical significance for helping entrepreneurs make correct decisions to reduce the losses caused by the pandemic.

## Introduction

The COVID-19 crisis broke out at the end of 2019 and became a pandemic on March 11, 2020, as the World Health Organization (WHO) declared. Since small- and medium-sized enterprises (SMEs) were heavily dependent on upstream and downstream supply chains, buyers and employees, they were severely affected by the pandemic (Etemad, 2020), and Chinese SMEs were no exception. According to a questionnaire survey conducted by the SMEs operation index research group of Postal Savings Bank of China on February 6 and 7, 2020, more than 90% of the SMEs delayed the start of business after the Spring Festival holiday. Nearly 80% of the SMEs’ performance deteriorated compared with the normal state (Zhang et al., 2020). Small, rapidly growing startups are particularly vulnerable to disruptions, including the generic risks peculiar to the entrepreneurial environment (Picken, 2017). Thus small- and micro-business entrepreneurs seem to suffer the most under the influence of the pandemic.

The personality approach is one of the classical and early approaches to entrepreneurship (Rauch and Frese, 2007). With the general acceptance of the five-factor theory of personality by researchers and the development of meta-analysis methods, the theory of personality has returned to the stage of entrepreneurial research in recent years (Zhao et al., 2010; Brandstätter, 2011). The entrepreneur is defined as the founder who owns and manages a small business (Zhao et al., 2010). The meta-analysis results showed that entrepreneurs’ extraversion, conscientiousness, emotional stability (low neuroticism), and openness to experience were significantly positively correlated with entrepreneurial performance, and openness appeared to be the most strong predictor of entrepreneurial performance among the five personality constructs (Zhao et al., 2010). But it is worth noting that most entrepreneurs, especially the more vulnerable entrepreneurs of small and micro enterprises, are more concerned about how to reduce losses in time in the face of the impact of the pandemic. During the great financial crisis (GFC) of 2008–2010, the proportion of innovating firms fell by around a third in the United Kingdom and took around four to 6 years to recover (Roper and Turner, 2020). Therefore, from the perspective of the personality approach, how to help entrepreneurs of small and micro businesses reduce their losses during the pandemic has become a problem worthy of attention.

Another key factor affecting performance is the process of strategic decision-making. Rajagopalan et al. (1993) developed an integrative framework of strategic decision-making processes by reviewing the past literature. Guo and Chen (2010) complemented this framework according to the research literature in the 15 years after 1993. They believed that the characteristics of the strategic decision process have a direct impact on the economic outcomes of strategic decisions. Furthermore, as one of the organizational factors, the characteristics of the top management team affect the strategic decision process and indirectly affect the economic outcomes. Strategic decision comprehensiveness is considered one of the most fundamental and enduring characteristics of strategic decision-making in an organization (Heavey et al., 2009). It is defined as the degree to which an organization is extensive, exhaustive and inclusive in gathering information from the external environment and making strategic decisions (Fredrickson and Mitchell, 1984; Forbes, 2007).

A systematic literature review conducted by Mendes et al. (2019) reviewed the relationship between personality and decision-making. However, little researches focused on the relationship between the Big Five personality, especially openness, and strategic decision comprehensiveness.

What’s more, there are two perspectives on the impact of strategic decision comprehensiveness on performance in an uncertain environment. One view is that environmental dynamism increases the benefits gained through comprehensiveness (Miller, 2008). And another perspective is that environmental dynamics decreases the benefits gained through comprehensiveness (Fredrickson and Mitchell, 1984), for the pursuit of comprehensiveness increases the cost. Thus a key question is whether strategic decision comprehensiveness enables firms to make better performance (or lose less) in the unstable environments caused by the pandemic.

The purpose of this study is to take entrepreneurs of SMEs as the research object and explore the influence of entrepreneurs’ openness personality on entrepreneurial performance during the COVID-19 pandemic and the special role played by strategic decision comprehensiveness. Given that most prior research on entrepreneurial performance focused on benefits, how to assist SMEs in reducing losses in the context of COVID-19 lockdown may be a unique contribution of this study as compared to other studies.

## Literature Review and Hypotheses Development

### Openness and Entrepreneurial Performance

Meta-analysis studies show that entrepreneurs’ Big Five personality traits are different from managers’ (Zhao and Seibert, 2006) and can predict their entrepreneurial intention and entrepreneurial performance (Zhao et al., 2010). Openness to experience appears to be the most strong predictor of entrepreneurs’ performance among the five personality constructs (Zhao et al., 2010).

Openness to experience is defined as “proactive seeking and appreciation of experience for its own sake, and as toleration for and exploration of the unfamiliar” (Piedmont, 1998). People high on openness can be described as “creative, innovative, imaginative, reflective, and untraditional” (Zhao and Seibert, 2006). The openness of individuals and teams is positively correlated with creativity (Schilpzand et al., 2011). To those high in openness, their creative ability has a positive linear relationship with accomplishments (King et al., 1996). For entrepreneurs, producing innovative products is very important to capture the market and grow the business. Therefore, entrepreneurs’ openness can positively predict their entrepreneurial performance.

On the other hand, openness is closely related to opportunity recognition. Studies have shown that genetic factors can largely explain the variance in opportunity recognition by influencing the probability that people are open to experience, and the phenotypic correlation coefficient between openness and opportunity recognition is 0.37 (Shane et al., 2010). Recognizing good opportunities is the beginning of the foundation of a new business. The small and medium-sized enterprises with high opportunity recognition ability tend to achieve higher firm performance through the innovation of the business model (Guo et al., 2017).

Hence, we posit the first hypothesis.

H1: Openness has a positive effect on entrepreneurial performance during COVID-19.

### Openness and Strategic Decision Comprehensiveness

According to strategic leadership theory, top managers’ field of vision, selective perception of information, and interpretation of information are affected by their values, cognitions, and personality (Cannella and Monroe, 1997). People high on openness can be characterized as intellectually curious and tending to seek novel ideas and embrace new experiences (Zhao and Seibert, 2006). According to the information-seeking theory of openness, people with higher openness/intelligence show general sensitivity to any type of new information; they are more sensitive to the rewarding value of information and are more motivated to seek out information (DeYoung, 2013, 2014). In addition, openness was positively correlated with update/monitoring in executive function, which is the ability to monitor and update information in working memory (Murdock et al., 2013). Strategic decision comprehensiveness just means the extent to which organizations want to search for more comprehensive and more information in the decision-making process.

Hence, we posit the second hypothesis.

H2: Openness is positively associated with strategic decision comprehensiveness.

### Strategic Decision Comprehensiveness and Entrepreneurial Performance

Based on previous literature, there are two contradicting perspectives on how the relationship between strategic decision comprehensiveness and firm performance is affected by environmental dynamics (Forbes, 2007).

The first view holds that environmental dynamism increases the benefits gained through comprehensiveness because unstable environments require collecting and analyzing a large amount of information. Then managers can improve their strategic understanding of the environment by being more comprehensive. Studies have shown that the comprehensiveness of strategic decision-making for family firms is positively associated with decision-making quality and firm performance (Carr et al., 2020). Miller (2008) study showed that, in a turbulent environment, both linear and non-linear relationships between comprehensiveness and performance are significantly positive. Specifically, comprehensiveness has a significant positive effect on performance, which is not significant in the case of low comprehensiveness.

The second view holds that environmental dynamics increase the cost of comprehensiveness and decrease the benefits gained through comprehensiveness. Because the pursuit of comprehensiveness increases the time and resources consumed by the decision-making process, the loss outweighs the gain. In a word, a company with low comprehensiveness is more suitable for an unstable environment. Its decision speed and flexibility allow for rapid, low-cost action to capture a changing set of opportunities that cannot be fully understood. Fredrickson and Mitchell (1984) found a consistently negative correlation between comprehensiveness and performance in an unstable industry environment. For new technology ventures, the strategic decision comprehensiveness of the top management team is significantly negatively correlated with financial performance (Souitaris and Maestro, 2009).

Therefore, we propose two competitive hypotheses:

H3a: Strategic decision comprehensiveness is positively associated with the entrepreneurial performance during COVID-19.
H3b: Strategic decision comprehensiveness is negatively associated with the entrepreneurial performance during COVID-19.

### Strategic Decision Comprehensiveness as a Mediator

In the process of enterprise management, strategic decision-making is closely related to the characteristics of top managers (Mendes et al., 2019) and firm performance (Guo and Chen, 2010; Yun, 2011) and always plays a mediating role. For example, the flexibility of strategic decision-making plays a mediating role between the Big Five personalities and enterprise performance (Nadkarni and Herrmann, 2010; Shalender and Yadav, 2019). And the positive effect of top management teams’ polychronicity on venture performance is partially mediated by strategic decision speed and comprehensiveness (Souitaris and Maestro, 2009).

Based on the above review of previous literature, we hypothesized that strategic decision comprehensiveness is a mediator between entrepreneurs’ openness and entrepreneurial performance during the pandemic. Since we have previously proposed two competing hypotheses about the relationship between strategic decision comprehensiveness and entrepreneurial performance, it is reasonable to speculate that there are also two competing hypotheses about the mediating role of strategic decision comprehensiveness. If strategic decision comprehensiveness positively impacts entrepreneurial performance, then strategic decision comprehensiveness plays a consistent mediating role. If strategic decision comprehensiveness negatively affects entrepreneurial performance, then strategic decision comprehensiveness may play an inconsistent mediating role, also known as the suppression effect.

Unlike consistent mediating effect, the sign of the direct and indirect effect of the independent variable on the dependent variable is opposite in suppression effect (MacKinnon et al., 2000). The relationship between the independent variable and the dependent variable is suppressed by the third variable (i.e., the inconsistent mediator variable). If the inconsistent mediator is not controlled, the regression coefficient between the independent variable and the dependent variable will become smaller or become an inverse relationship (Cohen et al., 2013).

Therefore, this paper proposes two competitive hypotheses:

H4a: Strategic decision comprehensiveness is a consistent mediator between openness and entrepreneurial performance during COVID-19.
H4b: Strategic decision comprehensiveness is an inconsistent mediator between openness and entrepreneurial performance during COVID-19.

## Materials and Methods

### Participant

Two hundred thirty-eight entrepreneurs from small and micro businesses were recruited from “wjx,” an online crowdsourcing platform in mainland China, which provides functions similar to Amazon Mechanical Turk. They completed the questionnaire between February 18, 2020, and February 26, 2020. At the time of our survey, the pandemic in the Chinese mainland had been effectively controlled, and all provinces had resumed work, production, and businesses, except for the worst-hit areas, such as Hubei province, where Wuhan is located. However, the severity of the pandemic situation and the policies for resuming work and production varied from place to place, and enterprises were faced with the dual pressure of pandemic prevention and control and resuming business operations to reduce losses.

### Measurement

#### Openness

Openness was measured by the Open-Mindedness subscale of the short form of the BFI-2 (the BFI-2-S) (Soto and John, 2017), which contains three facets with a total of six 5-point Likert items (1 = Disagree strongly, 2 = Disagree a little, 3 = Neutral, 4 = Agree a little, 5 = Agree strongly). The questionnaire and scoring information is provided in the Supplementary Material. A mean score of the six items (after reverse coding) was created as the indicator for openness. The higher the average score, the higher the openness level of the participants. The result of second-order confirmatory factor analysis showed that the Chinese version of the questionnaire had good structural validity (RMSEA = 0.070, CFI = 0.977, TLI = 0.943, SRMR = 0.039).

#### Strategic Decision Comprehensiveness

Strategic Decision Comprehensiveness was assessed by Miller et al. (1998) five 7-point Likert scale items (1 = Disagree strongly, 2 = Disagree, 3 = Disagree a little, 4 = Neutral, 5 = Agree a little, 6 = Agree, 7 = Agree strongly). The questionnaire and scoring information is provided in the Supplementary Material. A mean score was created as the indicator for strategic decision comprehensiveness. The result of confirmatory factor analysis showed that the Chinese version of this questionnaire had good structural validity (RMSEA = 0.054, CFI = 0.982, TLI = 0.965, SRMR = 0.027).

#### Entrepreneurial Performance During COVID-19 Pandemic

Participants were asked to rate on a scale of 1–10 for the entrepreneurial performance of their business before and after the COVID-19 pandemic. Then we got the score of entrepreneurial performance during the COVID-19 pandemic by subtracting the pre-pandemic score from the post-pandemic score, which ranged from −9 to 8. The higher the score, the better the entrepreneurial performance. Higher scores were mainly manifested as lower losses of entrepreneurial performance during the COVID-19 pandemic but also meant higher gains for a small group of participants.

#### Control Variables

Previous studies have demonstrated that gender (Coleman, 2016), age (Zhao et al., 2021), education level (Dickson et al., 2008), number of employees (Liu, 2010), entrepreneurial phase (Peng et al., 2018), entrepreneurial experiences (Tian and Xu, 2009), and the degree of home office realization of their businesses (Tønnessen et al., 2021) were relevant with performance. Thus we controlled these variables in the present research. The entrepreneurial experiences of the participants were also reported by answering in which year they started their business, and we subtracted the year they answered from 2020. The degree of home office realization of their businesses was measured by asking, “To what extent can your business achieve “home office”? Please use 1–10 to score.”

### Analyses

The reliability of constructs was assessed using Cronbach’s alpha with SPSS 22.0. And to examine the validity of the data, the measurement model was evaluated by confirmatory factor analysis (CFA) using Mplus 8.3 with the maximum likelihood estimation method. The composite reliability (CR) and average variance extracted (AVE) of the variables were calculated with the factor loadings. Given that openness has a second-order construct, we followed Nunkoo et al. (2017) in CFA analysis and used the factor loadings of the subscales of openness to calculate CR and AVE. Then, we used descriptive statistical analysis, correlation analyses and multiple regression analyses on the data with SPSS 22.0. Referring to Burton (2021), the assumptions of linear regression (non-multicollinearity, no autocorrelation, homoscedasticity and normality) were also tested for the regression models used SPSS 22.0. We inspected the variance inflation factors (VIFs) of all the regression models to check whether multicollinearity affected the data, applied the Durbin-Watson test to examine the no autocorrelation assumption. The scatterplots of the studentized residuals plotted against the unstandardized predicted values and the P-P plots of the regression models were constructed to test the assumption of homoscedasticity and normality, respectively. The PROCESS macro for SPSS (Hayes, 2017) was used to test the proposed mediation model.

## Results

The sample characteristics were presented in Table 1.

**TABLE 1:** Sample characteristics.

		Frequency	Percent (%)
Gender	Male	156	65.55
	Female	82	34.45
Education	Junior high school and below	7	2.94
	Senior high school	6	2.52
	Junior college	19	7.98
	Bachelor degree	168	70.59
	Master degree and above	38	15.97
Number of employees	10 or fewer employees	48	20.17
	10–19 employees	33	13.87
	20–29 employees	37	15.55
	30–39 employees	23	9.66
	40–49 employees	22	9.24
	50–99 employees	36	15.13
	100–299 employees	39	16.39
Entrepreneurial phase	The phase of foundation	53	22.27
	The phase of growth	142	59.66
	The phase of maturation	36	15.13
	The phase of transition	7	2.94
Entrepreneurial performance during COVID-19 pandemic	Post-pandemic entrepreneurial performance lower than before the pandemic	203	85.29
	Basically the same	17	7.14
	Post-pandemic entrepreneurial performance higher than before the pandemic	18	7.56

The Cronbach’s alpha of openness and strategic decision comprehensiveness (see Table 2) were both above the recommended threshold of 0.70. The results of CFA showed a good data fit (χ^2^ = 72.76, *df* = 40, *p* < 0.01, χ^2^/*df* = 1.82, CFI = 0.94, TLI = 0.91, RMSEA = 0.059, SRMR = 0.056). All items loaded significantly on the corresponding constructs. The AVE of openness was 0.76, while the AVE of strategic decision comprehensiveness was 0.34, below the recommended level of 0.5. According to Fornell and Larcker (1981), the AVE may be a more conservative estimate for the validity, and “on the basis of ρ_η_ (composite reliability) alone, the researcher may conclude that the convergent validity of the construct is adequate, even though more than 50% of the variance is due to error” (p. 46). As the CRs exceeded the suggested threshold value of 0.70 (Bagozzi and Yi, 1988) (see Table 2), the convergent validity of the construct can be adequate. Furthermore, the square roots of the AVE values were greater than the zero-order correlation coefficients between openness and strategic decision comprehensiveness (Zaiţ and Bertea, 2011).

**TABLE 2:** Results of the confirmatory factor analysis and validity test.

Construct	Item	Standardized loadings	CR	AVE	Cronbach’s α
Openness			0.90	0.76	0.73
Facet 1		0.67			
	opn1	0.71			
	opn4	0.80			
Facet 2		1.10			
	opn2	0.57			
	opn5	0.46			
Facet 3		0.78			
	opn3	0.58			
	opn6	0.78			
Strategic decision comprehensiveness			0.72	0.34	0.72
	sdc1	0.52			
	sdc2	0.55			
	sdc3	0.57			
	sdc4	0.63			
	sdc5	0.65			

*opn = openness, facet 1 = Aesthetic Sensitivity, facet 2 = Intellectual Curiosity, facet 3 = Creative Imagination; sdc = strategic decision comprehensiveness.*

Means, standard deviations, and correlations among the research variables in this study were analyzed and presented in Table 3.

**TABLE 3:** Means, standard deviations, and correlations among research variables.

Parameters	*M* ± *SD*	1	2	3	4	5	6	7	8	9
Gender (male = 1 female = 0)	–	–								
Age (years)	33.77 ± 6.29	0.07	–							
Education	3.94 ± 0.78	0.05	–0.08	–						
Realization of home office	5.24 ± 2.80	0.01	−0.13*	0.31***	–					
Number of employees	3.85 ± 2.17	0.03	0.04	0.29***	0.24***	–				
Entrepreneurial phase	1.99 ± 0.70	0.01	0.17**	0.01	–0.02	0.28***	–			
Entrepreneurial experiences (years)	5.82 ± 4.14	0.06	0.48***	−0.25***	−0.25***	0.02	0.32***	–		
Openness	3.84 ± 0.60	0.05	–0.09	0.27***	0.30***	0.22***	0.04	–0.05	–	
Strategic decision comprehensiveness	5.67 ± 0.66	–0.04	–0.05	0.13*	0.14*	0.31***	0.11	−0.13*	0.30***	–
Entrepreneurial performance during COVID-19 pandemic	–2.50 ± 2.32	–0.00	–0.08	–0.08	0.33***	0.00	0.04	0.00	0.21***	–0.10

*p < 0.05; **p < 0.01; ***p < 0.001.

The results of regression analysis were shown in Table 4. The highest VIF value for the regression models is 1.59 (presented in Table 4), which was less than 10, reflecting non-multicollinearity in the proposed model. The Durbin-Watson test value of the regression models ranged from 1.97 to 2.14 (presented in Table 4), which were very close to 2, indicating that there was little or no autocorrelation in the data and the residuals were independent of each other. The assumption of homoscedasticity and normality of the models were met according to the scatterplots of the studentized residuals plotted against the unstandardized predicted values (Supplementary Figures 1–3 presented in the Supplementary Material) and the P-P plots (Supplementary Figures 4–6 presented in the Supplementary Material).

**TABLE 4:** Multiple regression results of the mediation model.

	Entrepreneurial performance during COVID-19 pandemic (model 1)	Strategic decision comprehensiveness (model 2)	Entrepreneurial performance during COVID-19 pandemic (model 3)
**Control variables**			
Gender (male = 1 female = 0)	0.00	–0.05	–0.01
Age	–0.07	0.03	–0.07
Education	−0.20**	–0.04	−0.21**
Realization of Home Office	0.37***	–0.02	0.37***
Number of employees	–0.08	0.25***	–0.03
Entrepreneurial phase	0.05	0.08	0.07
Entrepreneurial experiences	0.07	−0.17*	0.04
**Independent variable**			
Openness	0.17*	0.25***	0.21**
**Mediator**			
Strategic Decision Comprehensiveness			−0.18**
**Model fit statistics**			
*F*	6.34***	6.12***	6.66***
*R* ^2^	0.18	0.18	0.21
VIF	1.55	1.55	1.59
Durbin-Watson test value	1.99	2.14	1.97

Standardized regression coefficient were shown. **p < 0.01; ***p < 0.001.

After controlling for participant’s gender, age, education level, number of employees, entrepreneurial phase, entrepreneurial experiences, and the degree of home office realization of their businesses, the total effect of openness on entrepreneurial performance during the COVID-19 pandemic (model 1) was significantly positive (β = 0.17, *t* = 2.56, *p* < 0.05). Hypothesis 1 was supported.

**FIGURE 1:**
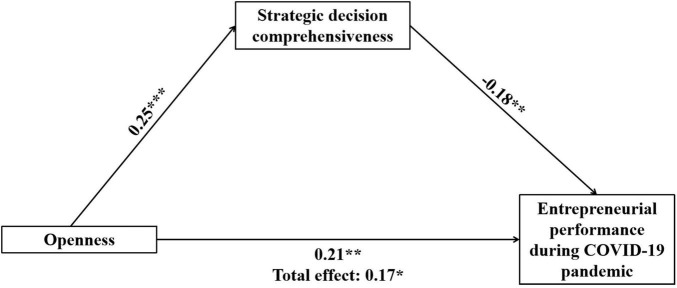
The mediation model with standardized regression coefficients. *p < 0.05; **p < 0.01; ***p < 0.001.

On this basis, we further tested the mediating effect of strategic decision comprehensiveness (see Figure 1). As shown in Table 4, the effect of openness on strategic decision comprehensiveness (model 2) was significantly positive (β = 0.25, *t* = 3.85, *p* < 0.001). Hypothesis 2 was supported. The effect of strategic decision comprehensiveness on entrepreneurial performance during COVID-19 pandemic (model 3) was significantly negative (β = –0.18, *t* = −2.78, *p* < 0.01). Thus, Hypothesis 3b was supported. The direct effect of openness on entrepreneurial performance during COVID-19 pandemic after controlling the mediator (model 3) was also significantly positive (β = 0.21, *t* = 3.19, *p* < 0.01).

Then, we applied Model 4 with 5,000 bootstrap samples in PROCESS macro to examine the mediation effect. The result showed that 95% bootstrap confidence intervals of the completely standardized indirect effect did not include zero [*Effect* = −0.05, 95% Boot CI = (−0.095, −0.002)]. The indirect effect was significantly negative, and the standardized direct effect was significantly positive [*Effect* = 0.21, 95% Boot CI = (0.002, 0.314)]. The sign of the direct and indirect effect was opposite, and the direct effect was larger than the total effect, indicating that strategic decision comprehensiveness played an inconsistent and partial mediating role between openness and entrepreneurial performance during COVID-19 pandemic. Therefore, Hypothesis 4b was supported.

## Discussion

### General Discussion

Taking entrepreneurs of SMEs as research objects, this study explores the impact of entrepreneurs’ openness personality on their entrepreneurial performance during the COVID-19 pandemic and the inconsistent mediating role of strategic decision comprehensiveness.

The research result showed that entrepreneurs’ openness had a significantly positive impact on their entrepreneurial performance, consistent with previous research results (Zhao et al., 2010; Araujo-Cabrera et al., 2016). People with high openness tend to come up with novel ideas, be more creative, and find alternative values and ideas more easily. They have high flexibility in strategic decision-making, which often enables them to improve performance in such alternate competitive and technologically changing environments (Shalender and Yadav, 2019). Garretsen et al. (2020) also showed that cities with higher levels of openness tended to be more resilient during the national recession in the United Kingdom.

The relationship between the openness of entrepreneurs and strategic decision comprehensiveness was significantly positive. To our knowledge, this is the first time that the openness personality trait has been found to relate to strategic decision comprehensiveness. This may be due to the high openness individuals are more motivated to seek out information and are more sensitive to any type of new information and their rewarding value (DeYoung, 2013, 2014). A facet of openness, trait curiosity, was also positively associated with information-seeking (Jach and Smillie, 2020).

Another critical question this study focused on was whether strategic decision comprehensiveness enables firms to make better performance in unstable environments. The research result supported H3b rather than H3a of the two competitive hypotheses in this study. That is, the relationship between strategic decision comprehensiveness and performance was significantly negative. This result can be explained by the model proposed by Forbes (2007). He presented that, environmental instability which was also used as environmental dynamism (Heavey et al., 2009) and turbulence (Miller, 2008) in previous researches, should be divided into two conditions, uncertainty and ambiguity, according to the “quantity” and “determinacy” of the available information in the organizational information environment.

Uncertainty means that decision-makers know a range of possible outcomes and the probability of each outcome. And the “quantity” and “determinacy” of available information, which are the two key dimensions of the organizational information environment, are both high in the condition akin to uncertainty (Forbes, 2007). Therefore, the organizational information environment is unstable but analyzable, so more information is helpful in making strategic decisions to improve overall performance. However, in the case of ambiguity, decision-makers are uncertain about the probabilities of outcomes and the processes leading to those outcomes. At least one of the “quantity” and “determinacy” of available information is relatively low in conditions akin to ambiguity (Forbes, 2007). Thus the environment is not analyzable, and decision-makers are less likely to gather and analyze information (Dean and Sharfman, 1993; Sharfman and Dean, 1997; Shepherd and Rudd, 2014). Pursuing a high quantity of information costs a lot of time and resources and is likely to lead to misdirection. Therefore, strategic decision-making comprehensiveness will have a negative impact on performance.

The object and background that this study focused on just match the characteristics of ambiguity. On the one hand, The amount of information may exponentially in a short period because of a specific incident such as the COVID-19 pandemic. In this so-called “infodemic” situation, misinformation and rumors, as well as information with questionable intentions that can be manipulated, keep popping up (Garcia and Duarte, 2020; Zarocostas, 2020). And this phenomenon can be amplified and spread further and faster through social networks, just like the virus (Zarocostas, 2020). Therefore, even if a large amount of information can be obtained, when the accuracy of the information cannot be ensured, excessive or even contradictory information will increase the burden of information assessment and decision-making. On the other hand, the investigation was at the beginning of China’s resumption of work and production. The severity of the pandemic varies in different regions, and the policies for the resumption of work and production were not consistent. The upstream and downstream industrial chains and product sales had been greatly affected (Lu et al., 2020). Besides, the pandemic had the possibility of a rebound at that time. Therefore, the uncertainty of information obtained by small- and micro-businesses entrepreneurs was high, and decision-makers were unable to make comprehensive and thoughtful decisions. The higher the comprehensiveness of their strategic decisions, the higher the costs of the decision process (time and resources), and the more likely it was to bring negative effects.

Furthermore, the results supported H4b of the two competitive hypotheses, namely, the strategic decision comprehensiveness played an inconsistent mediating role between openness and entrepreneurial performance during the pandemic. On the one hand, high openness had a direct and positive impact on entrepreneurial performance during the pandemic period. On the other hand, individuals with low openness could also reduce their time losses through incomprehensive and rapid strategic decisions.

### Theoretical Implications

First, this study demonstrates the positive effect of entrepreneurs’ openness on strategic decision comprehensiveness. To our knowledge, the openness personality trait has been found to associate with strategic decision comprehensiveness for the first time. Secondly, it provides more empirical data to support the negative impact of strategic decision comprehensiveness on entrepreneurial performance in the context of uncertainty and unanalyzable information. The entrepreneurial performance was measured by comparing before and after the pandemic to make the results more convincing. Finally, the study demonstrated the inconsistent mediating effect of strategic decision comprehensiveness between entrepreneurs’ openness and entrepreneurial performance during the pandemic.

### Practical Implications

Firstly, considering that high openness is one of the personality characteristics that distinguishes entrepreneurs from managers (Zhao and Seibert, 2006), entrepreneurship education can focus on cultivating entrepreneurs’ openness personality. Even in the face of an unstable environment, high openness entrepreneurs can show their advantages.

In addition, when faced with sudden and uncertain dangerous situations such as the COVID-19 pandemic, entrepreneurs with high openness need to give full play to their advantages of being creative and able to identify opportunities. However, entrepreneurs with low openness are not sensitive to the reward value of information. They don’t need to make great efforts to search for more information and should make decisions quickly instead of thinking ahead and backward.

### Limitation

This study is a cross-sectional study, and since the data is collected through the online crowdsourcing platform, it is not easy to track the entrepreneurs we recruited. However, future studies should consider using longitudinal data to examine the inconsistent mediating role of the comprehensiveness of strategic decisions, especially for firms recovering from COVID-19.

Furthermore, entrepreneurial performance before and after the COVID-19 pandemic was assessed by the subjective evaluation of entrepreneurs. Although the subjective evaluation method has good discrimination validity for measuring entrepreneurial economic return (Chen et al., 2014), it is also valuable to apply the other objective indicators that can be used to measure the entrepreneurial performance and economic recovery across industries.

Finally, previous studies have shown that decision-makers’ risk-taking propensity and proactiveness are important moderating variables between strategic decision comprehensiveness and corporate entrepreneurship (Heavey et al., 2009). Subsequent studies can further explore their moderating effects on the mediation model in the present study.

## Conclusion

The present research extends prior literature by investigating the impact of entrepreneurs’ openness personality trait on entrepreneurial performance during the COVID-19 pandemic, as mediated by strategic decision comprehensiveness. Our evidence highlights the positive effect of openness on strategic decision comprehensiveness and the negative relationship between strategic decision comprehensiveness and entrepreneurial performance in the unstable and unanalyzable organizational environment. Furthermore, we demonstrate the inconsistent mediating role of strategic decision comprehensiveness. No matter whether the openness of entrepreneurs is high or low, they can reduce the losses caused by the pandemic by adopting strategies suitable for them. Moreover, the negative impact of strategic decision comprehensiveness on enterprise recovery after the pandemic may be of particular concern.

## Data Availability Statement

The raw data supporting the conclusions of this article will be made available by the authors, without undue reservation.

## Ethics Statement

The studies involving human participants were reviewed and approved by Ethical Review Board of the Institute of Psychology, Chinese Academy of Sciences. Written informed consent from the participants’ legal guardian/next of kin was not required to participate in this study in accordance with the national legislation and the institutional requirements.

## Author Contributions

MZ and JX developed the research project. WM, JX, and FL carried out the data collection. WM, SL, and XL carried out the data analysis. WM wrote the first draft. WM and MZ revised the manuscript. All authors contributed to the article and approved the submitted version.

## Conflict of Interest

The authors declare that the research was conducted in the absence of any commercial or financial relationships that could be construed as a potential conflict of interest.

## Publisher’s Note

All claims expressed in this article are solely those of the authors and do not necessarily represent those of their affiliated organizations, or those of the publisher, the editors and the reviewers. Any product that may be evaluated in this article, or claim that may be made by its manufacturer, is not guaranteed or endorsed by the publisher.
