# Supplementary material for: Openness and Entrepreneurial Performance During COVID-19 Pandemic: Strategic Decision Comprehensiveness as an Inconsistent Mediator
Source: Front Psychol. 2022 Jan 13;12:806756. doi: 10.3389/fpsyg.2021.806756 (PMC8793850; doi:10.3389/fpsyg.2021.806756)
Supplement: Supplementary file 1 [file Data_Sheet_1.PDF]

Supplementary Material

1 Measurement of openness

Here are a number of characteristics that may or may not apply to you. For example, do you agree that you are someone who likes to spend time with others? Please select an option after each statement indicating the extent to which you agree or disagree with the statement.

Disagree strongly	Disagree a little	Neutral	Agree a little	Agree strongly
1	2	3	4	5

**I am someone who. . .**	facets
1. Is fascinated by art, music, or literature.	Aesthetic Sensitivity
2. Has little interest in abstract ideas. (R)	Intellectual Curiosity
3. Is original, comes up with new ideas.	Creative Imagination
4. Has few artistic interests. (R)	Aesthetic Sensitivity
5. Is complex, a deep thinker.	Intellectual Curiosity
6. Has little creativity. (R)	Creative Imagination

Note. (R) = Reverse Scored Item.

Reference: Soto, C. J., & John, O. P. (2017). Short and extra-short forms of the Big Five Inventory–2: The BFI-2-S and BFI-2-XS. *Journal of Research in Personality*, *68*, 69-81. https://doi.org/10.1016/j.jrp.2017.02.004

2 Measurement of strategic decision comprehensiveness

The following are some practices to take when confronted with an important, non-routine problem or opportunity. Please choose the degree of your consent to the following practices according to the situation of your entrepreneurial team.

Disagree strongly	Disagree	Disagree a little	Neutral	Agree a little	Agree	Agree strongly
1	2	3	4	5	6	7

My firm ……

1. develop many alternative responses

2. consider many diverse criteria for eliminating possible courses of action

3. thoroughly examine multiple explanations for the problem or opportunity

4. conduct multiple examinations of any suggested course of action

5. search extensively for possible responses

Reference: Miller, C. C., Burke, L. M., & Glick, W. H. (1998). Cognitive diversity among upper-echelon executives: implications for strategic decision processes. *Strategic Management Journal*, *19*(1), 39-58. https://doi.org/10.1002/(SICI)1097-0266(199801)19:1<39::AID-SMJ932>3.0.CO;2-A

3 The scatterplots of the studentized residuals plotted against the unstandardized predicted values

The scatterplots of the studentized residuals plotted against the unstandardized predicted values showed a horizontal band, indicating that the regression equation satisfied the assumption of homoscedasticity.

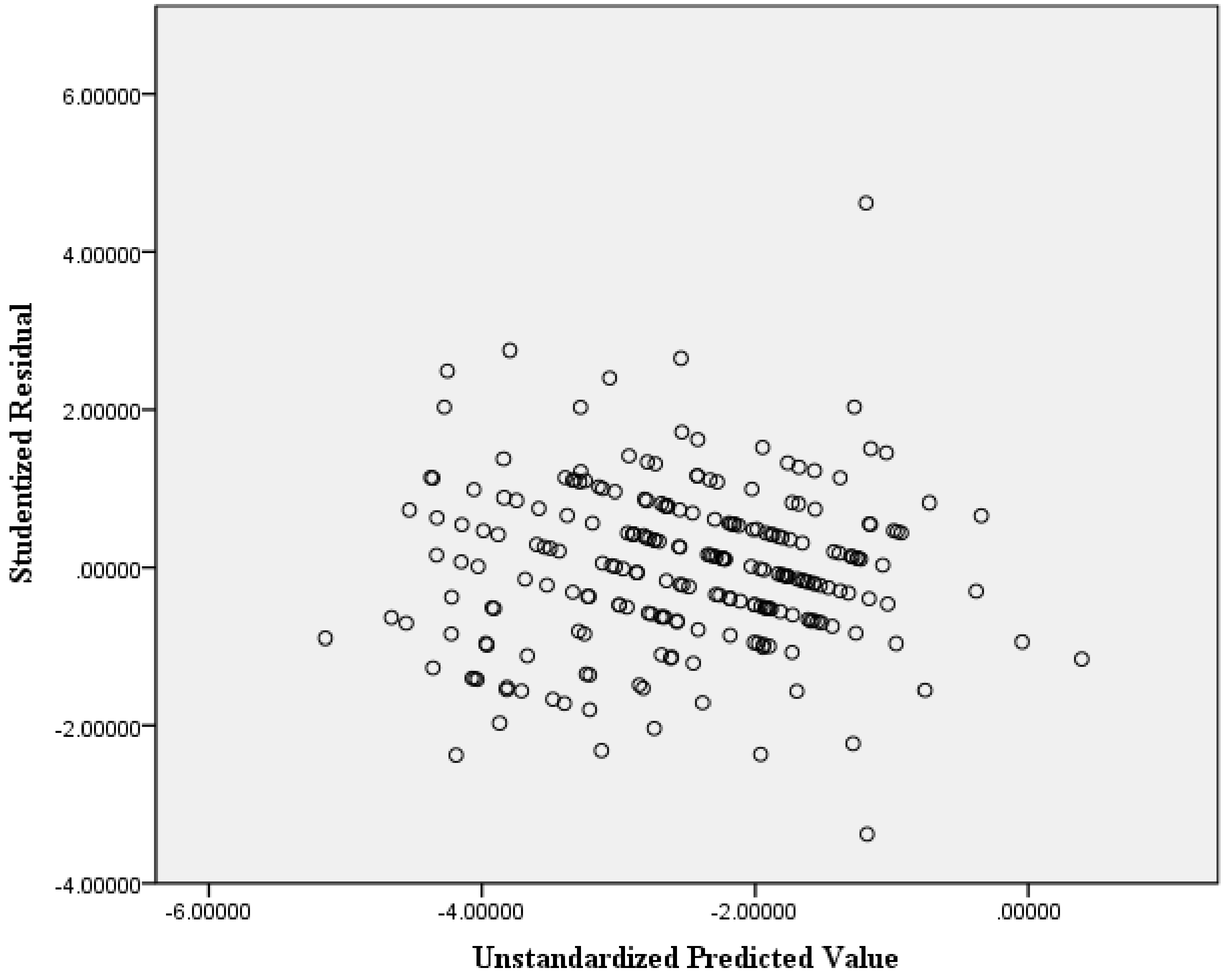

Supplementary Figure 1. The scatterplot of the studentized residuals plotted against the unstandardized predicted values for model 1 in Table 4.

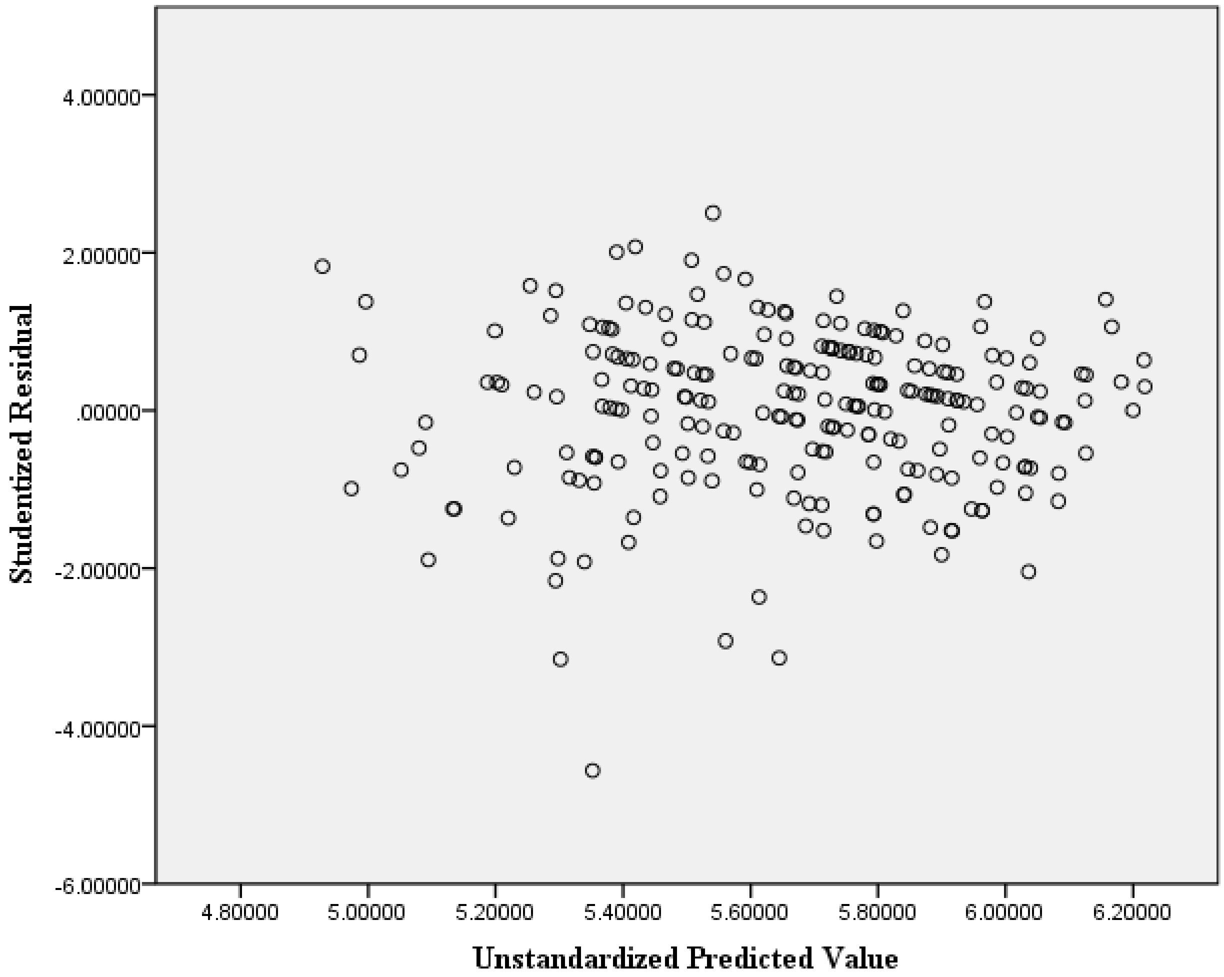

Supplementary Figure 2. The scatterplot of the studentized residuals plotted against the unstandardized predicted values for model 2 in Table 4.

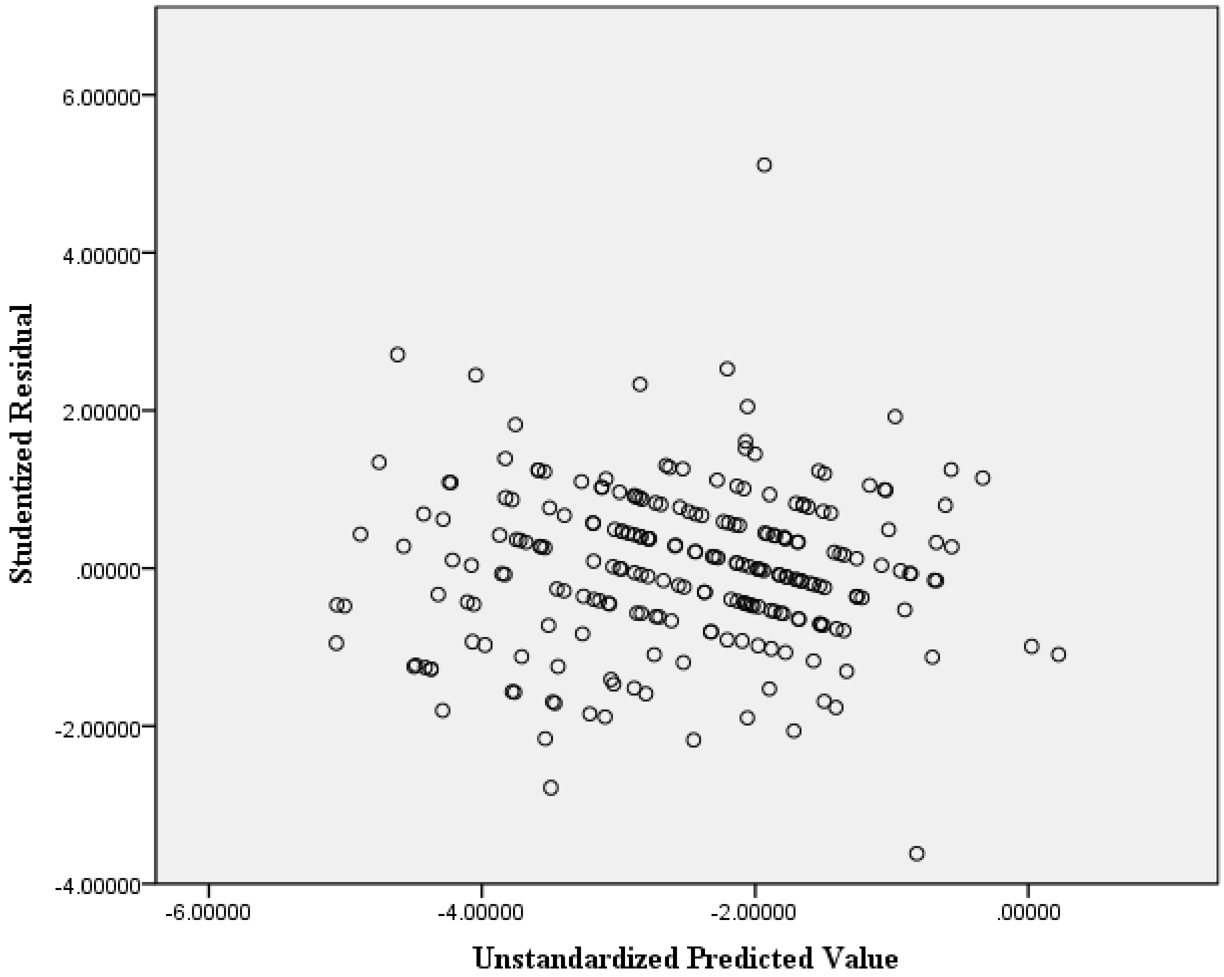

Supplementary Figure 3. The scatterplot of the studentized residuals plotted against the unstandardized predicted values for model 3 in Table 4.

4 P-P plots

The P-P plot showed that the points on the plot roughly form a straight diagonal line, thus the residuals were normally distributed and the normality assumption was met.

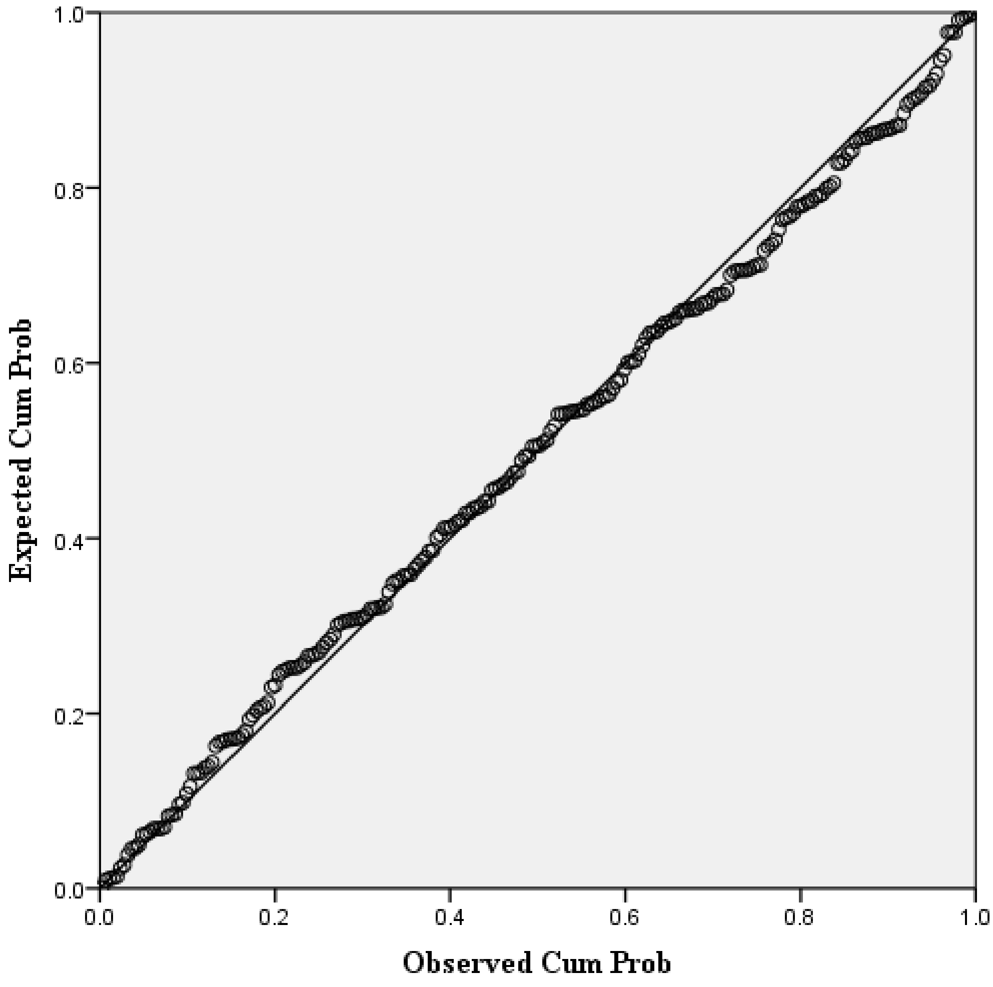

Supplementary Figure 4. The P-P plot for model 1 in Table 4.

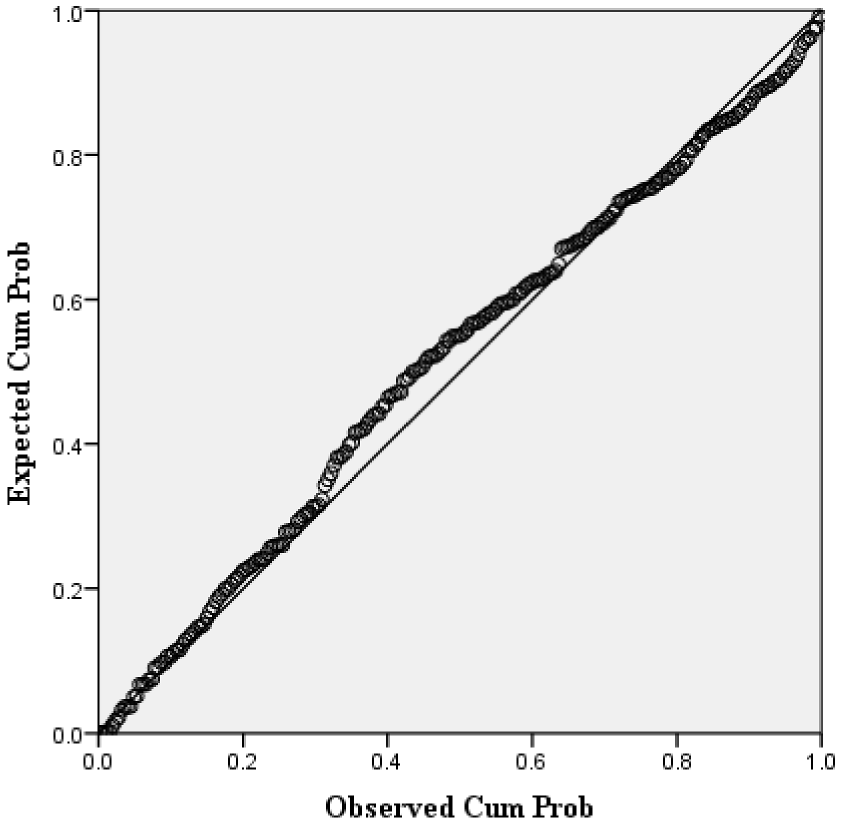

Supplementary Figure 5. The P-P plot for model 2 in Table 4.

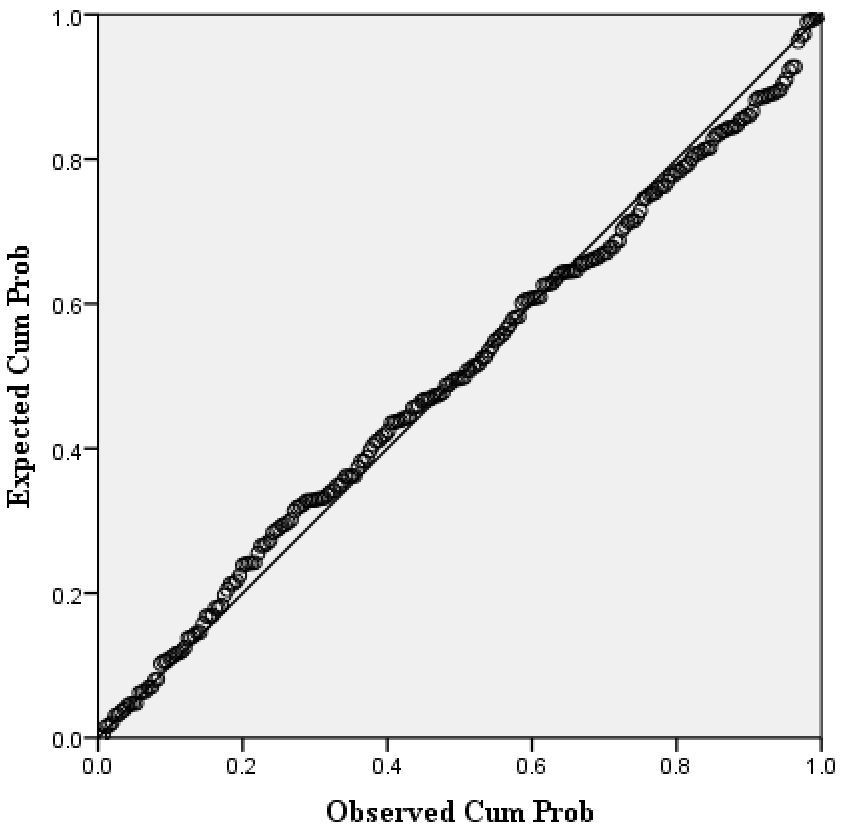

Supplementary Figure 6. The P-P plot for model 3 in Table 4.
